# Robert Braidwood (Bob) Sim. 1951–2021: A Disciple’s Perspective

**DOI:** 10.3390/v13061111

**Published:** 2021-06-10

**Authors:** Álvaro Díaz

**Affiliations:** 1 Área Inmunología, Departamento de Biociencias, Facultad de Química, Universidad de la República, Montevideo 11600, Uruguay; adiaz@fq.edu.uy; 2 Instituto de Higiene, Montevideo 11600, Uruguay

Three and a half months ago, we were very much saddened by the news of Bob Sim’s passing away. I will attempt to portray Bob from the point of view of a disciple; I was his postgraduate student between 1994 and 1997. At that time, Bob was about one-third into his mentoring career, during which he supervised or co-supervised over 30 postgraduate students. 

As context to my personal account, I will first attempt to give a short summary of Bob’s personal background and career. Bob grew up in rural Scotland. He certainly did not come from a privileged background, and if he had access to higher education, it was at least partly because he excelled at every stage in his educational trajectory. He obtained his first degree in Biochemistry from the University of Edinburgh in 1973, which is also where he met his lifelong partner Edith. He obtained his Doctorate (D.Phil.) from the University of Oxford in 1976. His thesis was on C1, the first component of the complement system. Bob’s supervisor during his D.Phil. was Ken Reid, later chosen as a Fellow of the Royal Society for his contributions to the elucidation of the structure of C1. During his D.Phil., Bob also received informal tuition from Rodney Porter, by then already Nobel laureate for the elucidation of the structure of antibodies. Porter had in turn been the first doctoral student of Frederick Sanger, twice Nobel laureate for the sequencing of proteins and DNA. From this highly distinguished scientific ancestry, Bob absorbed everything that was useful knowledge, without developing any urge to seek similar laurels for himself; his very nature appeared to make him immune to such anxieties. I will come back to these aspects later, but Bob was centred in duty and hard work, curiosity about nature, and empathy for others. 

After his D.Phil, Bob worked for 2 years in Grenoble, France, as postdoc under Maurice Colomb, still on the subject of C1. In 1978, he went back to Oxford, to lead a research group at the MRC Immunochemistry Unit, directed by Porter and later by Ken Reid. Bob was one of the pillars of the Unit until its closure in 2008. 

Bob’s scientific career was centred on the biochemistry of the proteins of complement, and the functionally related collectins. In all, he published about 350 papers, some of which have been cited over 1000 times. It is difficult to highlight a single contribution by Bob. Perhaps his contributions with more impact were the first purification of the non-activated form of C1 (during his D.Phil.), the characterisation of the reaction by which C3 binds to surfaces, structural studies of factor H, the identification of autoantibodies against C1 inhibitor as the cause of the disease autoimmune angioedema (with John Jackson from Dublin), the identification of what is probably the main cellular receptor for C1, the discovery that antibodies decorated with aberrant sugars activate complement and how they do so, and the analysis of complement activation by carbon nanotubes. 

My first contact with Bob was via fax. It was 1992 and my country, Uruguay, was only seven years into re-gained democratic rule after a long dictatorship that had all but killed local science. In our rather provincial context and before the expansion of e-mail, the fax machine was a magic periscope of sorts, as it allowed us to establish agile contact with scientists abroad. Within the busy research group led by Alberto Nieto, I was an M.Sc. student baffled by the practical difficulties of quantifying mouse C3 using a home-grown antiserum. Ana Ferreira, in our lab and also working on complement, suggested me to write to Bob for advice. Ana had visited Bob in Oxford in 1991, having contacted him thanks to Valerie Dee, an Anglo-Uruguayan D.Phil. student in Bob’s lab. Receiving Bob’s long faxed response message was an unforgettable event. There I had, from this international authority in complement, all the detailed practical advice I could hope for in the most down-to-earth tone imagined. Just wonderful. 

Thanks to a European Community joint research grant obtained by Ana and Bob, I became Bob’s D.Phil. student. Whereas in Uruguay at the time long discussions and intellectual jousts were highly valued, as a new student in Bob’s lab, I quickly felt under pressure to get on with experimental work, every day, no procrastination allowed. After some three months, I started to realise that under Bob’s reasonable pressure and highly practical advice, my experimental work would move forward in a way that I had not experienced before. So whatever stress I initially felt gave way to a new feeling of professional self-esteem based on concrete gradual achievements, and also to a great deal of admiration for my supervisor. Over the following years, I rounded off my understanding of Bob’ leadership: a leadership rooted in a sober but exceptionally strong personality, in duty and compassion as major values, and in a distinct love for science in its most tangible manifestations. 

Bob had a tiny office next to the lab, the door of which was rarely closed (Figure 1). When he walked past us as we worked at the bench, he would emit a characteristic soft hum, as if saying “I’m here. I’m supervising, and my kind of supervision is friendly”. No student wished to be “told off” by Bob. He would never raise his voice, but a few strongly emphasised words from Bob were enough to bring any student back into line. Yet Bob was highly liked by his students; the atmosphere in his lab was happy and friendly, as it happens in workplaces when the bosses are just. “Thank you for the hard work” was Bob’s reply when we thanked him for the pub lunches to which he would often invite us.

Whenever the occasion was right, Bob would share with us some of his endless store of anecdotes. Never motivated by self-aggrandizement, Bob’s selection of anecdotes was instead shaped by his all-encompassing interest in how the world functioned and a predilection for subtly comic contradictions, which he expressed with understated sense of humour. 

Bob’s empathy for others was evident in his painstaking teaching, using writing as the major means of communication, to a student newly arrived from China with essentially no oral English. Furthermore, in his unfaltering support of a student that had to endure a serious medical problem, or in him opening the lab to an intellectually restless and very pleasant senior scientist who was long past his retirement age. Bob was very distinctly supportive towards his younger collaborators’ careers; he invariably helped his students organise their careers—academic or otherwise—past their stage in his lab. Bob was also, for 25 years, extraordinarily generous towards our extended research group in Uruguay, selflessly offering reagents, encouragement and scientific advice. For his contribution to the development of immunology in Uruguay, the School of Chemistry of our national University awarded Bob an honoris causa Doctorate in 2017 (Figure 2).

Bob’s love for science was a part of his passion to understand how the world functions. His practice of science was rooted in a quiet confidence in his capacity to operate on the material world around him. I imagine that this confidence stemmed from the practice of manual tasks as a child in his various rural homes. Certainly, this kind of confidence, in addition to intellect, must have been at play when, being a D.Phil. student and short of money, he worked during holidays at the Tay Salmon Fisheries, and was chosen by his fellow workers as foreman. The passion for understanding meant that Bob followed students’ bench work very closely, analysing each result with genuine interest and curiosity. With Bob as mentor, results ruled over any previous ideas, as it should be. Bob’s practical nature meant that his advice was not only highly adapted to reality, but also supported by his direct participation in the trickier procedures. It was normal to see Bob engrossed in organising the lab’s cold room or freezer space, together with Beryl Moffatt, who was Bob’s long-time technician and for a few years also his M.Sc. student. 

Following the closure of the MRC Immunochemistry Unit, Bob worked, and shared his unique collection of reagents, with a range of scientists in Pharmacology in Oxford, at Kingston University, and with Wilhelm Schwaeble in Leicester. He also collaborated extensively with Uday Kishore at Brunel University. Despite the fact that the Unit always held a special place in his affections, Bob adapted to the circumstances. His stoicism, courage, and thankfulness for the life he had were never more obvious than when he dealt with cancer in 2012, and during his final battle with a second unrelated cancer in 2020. 

I like to think that Bob’s legacy, as much as in the discoveries in which he participated, is in the values that he transmitted to those who were his disciples; sense of duty and care for other people, applied to scientific mentoring in particular. Within the sometimes tough world of competitive science, a legacy of decency at its highest level. 

## Figures and Tables

**Figure 1:**
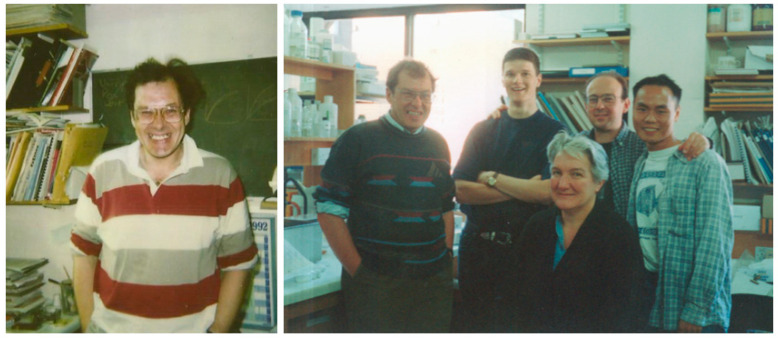
Bob in his office (**left**) and in the lab (**right**) ca. 1996. In the right-hand photograph, from left to right: Bob, Guy Stuart (D.Phil. student), Beryl Moffatt, the author, and Niki Wong (D.Phil. student); Bing Bin Yu and Timothy Hickling (not in the picture) were also D.Phil. students in the group at the time.

**Figure 2:**
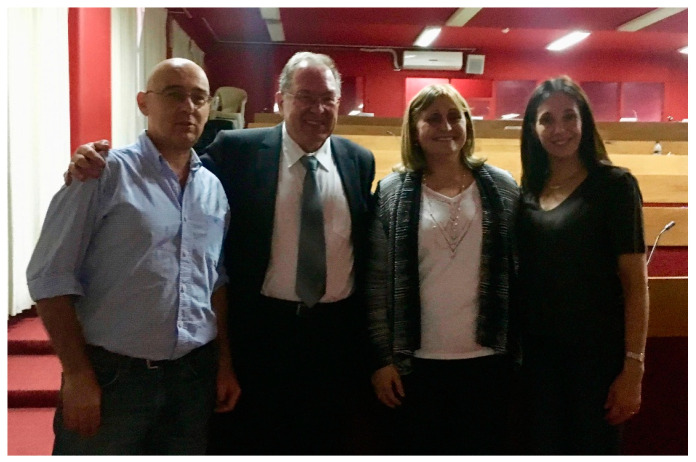
Honoris causa Doctorate in Uruguay, 2017. From left to right: the author, Bob, Maria H. Torre (then Dean of the School of Chemistry), and Ana María Ferreira.

